# Adnexal lesions after hysterectomy: A retrospective observational study

**DOI:** 10.4274/jtgga.galenos.2018.2018.0051

**Published:** 2019-08-28

**Authors:** Ayşegül Öksüzoğlu, Şebnem Özyer, Özlem Yörük, Rıfat Taner Aksoy, Ömer Hamit Yumuşak, Özlem Evliyaoğlu

**Affiliations:** 1 Clinic of Obstetrics and Gynecology, Gynecology and Endoscopic Surgery Unit, University of Health Sciences, Ankara Dr. Zekai Tahir Burak Women’s Health Training and Research Hospital, Ankara, Turkey

**Keywords:** Adnexal lesion, adnexal preservation, hysterectomy, re-operation

## Abstract

**Objective:**

To characterize adnexal lesions detected in patients who had undergone previous hysterectomy with one or both ovaries conserved, and to define the clinical, pathologic, and surgical characteristics of the adnexal lesions in these patients.

**Material and Methods:**

A retrospective observational study was conducted on patients who had undergone a previous abdominal hysterectomy with one or both adnexa preserved and who had subsequently presented with an adnexal lesion. Characteristics of lesions, operative, and pathologic findings in patients who required a re-operation were noted.

**Results:**

One hundred thirty-seven patients presented with an adnexal lesion after hysterectomy. Of the 137 patients, 71 (51.8%) had undergone a re-operation (re-operated group), the rest of the patients (n=66, 48.1%) remained on follow-up (follow-up group) in whom the lesion disappeared during follow-up period. Adnexal lesions that were re-operated were significantly larger (p<0.001), more complicated (p=0.04), and had more septations (p=0.01) than in the follow-up group. The origin of the adnexal lesion was confirmed as the ovary in 59 (83%) patients, and as the peritoneum in 8 (11.2%) patients during surgery. All of the adnexal lesions arising after hysterectomy and required a re-operation were confirmed to be benign.

**Conclusion:**

Almost half of the lesions detected after hysterectomy disappeared during the follow-up period. The adnexal lesions that were re-operated were more symptomatic, larger, and had more complicated lesions. All lesions that were re-operated were found to be benign, mostly originating from the ovary.

## Introduction

Hysterectomy is one of the most commonly performed surgical procedures among women (1), and the majority of these are performed for benign diseases of the uterus (2). There is debate about hysterectomy for benign conditions regarding the performance of concurrent prophylactic adnexal surgeries, oophorectomy or salpingectomy. The major benefits of prophylactic salpingo-oophorectomy are the prevention of subsequent ovarian and breast cancer, and the reduction in the risk of future adnexal surgery (3). However, oophorectomy is associated with a number of potential risks in the long term related with earlier surgical menopause in premenopausal women who face risks of cardiovascular disease, osteoporosis and hip fractures, neurologic and psychiatric disorders, and colorectal and lung cancers (3). The benefits must be weighed against these potential adverse effects during preoperative patient counseling and decision making. Recent studies investigating the trends of adnexal surgeries at the time of hysterectomy for benign indications suggested that the rate of ovarian preservation in women younger than 50 years of age was increasing (2,4).

On the other hand, there is a risk of repeat adnexal surgery for *de novo* developed adnexal pathologies after hysterectomy when adnexa are retained. These adnexal lesions pose a challenge for gynecologists as well as the patients. The incidence of subsequent surgery after hysterectomy varies from 2.8-9.2% (5). In a cohort study of 2561 hysterectomies over 20 years, during which one or both ovaries were preserved, Dekel et al. (6) found that the most common indications for reoperation were pelvic pain (71.3%) and presence of an asymptomatic adnexal mass (24.6%).

There are a limited number of studies in the literature investigating adnexal lesions arising after hysterectomy (6,7,8,9,10,11,12,13,14). Within this scope, the aim of the study was to characterize adnexal lesions detected in patients who had undergone previous hysterectomy with one or both ovaries conserved, and to define the clinical, pathologic, and surgical characteristics of the adnexal lesions in these patients.

## Material and Methods

A retrospective observational study was conducted at the Gynecology and Endoscopic Surgery Unit of Ankara Dr. Zekai Tahir Burak Women’s Health Training and Research Hospital. The study was approved by the Institutional Review Board of the hospital (approval no: 2014/47). Patients who were eligible for the study were those with a previous abdominal hysterectomy for benign indications with one or both adnexa preserved during the years of 2007-2013, and had subsequently presented with an adnexal pathology. Patients who had undergone vaginal or laparoscopic hysterectomy were not included in the study. Clinical, pathologic, and surgical data related with the hysterectomy procedure and adnexal pathology were retrospectively collected from the medical records of the patients. Demographic characteristics including ages and hysterectomy details of the patients, characteristics of adnexal lesions including symptoms, interval to diagnosis, re-operation and follow-up, serum E2 and CA125 levels on detection, location, ultrasonographic features, operative and pathologic characteristics of adnexal lesions in patients who required a re-operation including type of the procedure, origin of the adnexal lesion, malignancy rate, operative complications, and length of hospital stay were noted.

Statistical analyses were performed using the SPSS statistics package (version 16.0; SPSS Inc, Chicago, IL). The Kolmogorov–Smirnov test was used for the normality testing of the data sets. The comparison of continuous variables between the groups was performed through Student's t-tests or Mann-Whitney rank sum tests, and the chi-square test was used for categorical variables. Differences with p<0.05 were considered to be statistically significant.

## Results

Between 2007 and 2013, a total of 3566 abdominal hysterectomies were performed for benign indications in our institution. In 619 of these hysterectomies, at least one of the adnexa was saved. Among these patients who were available for follow-up in our institution, 137 (22.1%) presented with an adnexal lesion during the follow-up period. Of the 137 patients with an adnexal lesion, 71 (51.8%) had undergone a re-operation (re-operated group), and among the re-operated group, 7 (9.8%) of them had undergone a second procedure other than hysterectomy due to the adnexal pathology. The rest of the patients (n=66, 48.1%) remained on follow-up (follow-up group). The adnexal lesions detected in this group disappeared during the follow-up period. The mean age of patients at the time of hysterectomy who were later diagnosed as having adnexal lesion was 46.4±4.0 years (Table 1). The most common surgical indication for the previous hysterectomy was leiomyoma (n=94, 68.6%) (Table 1). Table 2 demonstrates the characteristics of the adnexal lesions in the re-operated and the follow-up groups. The number of symptomatic patients were statistically higher in the re-operated group (p=0.012). The median interval between the hysterectomy and the diagnosis of adnexal pathology was 31 (minimum 1, maximum 216) months in the re-operated group and 4 (minimum 1, maximum 56) months in the follow-up group (p<0.001). There were no significant differences with respect to serum E2 and CA125 levels. The mean size of the adnexal lesion was 71.3±25.2 mm in the re-operated group, whereas it was 44.0±10.9 mm in the follow-up group. Mural nodules, septations inside the adnexal lesion and abnormal Doppler findings were detected in 7 (9.9%), 48 (67.6%), 3 (4.2%) patients in the re-operation group, and 4 (6.1%), 31 (47.0%), 1 (1.5%) patients in the follow-up group, respectively. Adnexal lesions that were re-operated were significantly larger (p<0.001), more complicated (p=0.04), and had more septations (p=0.01) than in the follow-up group. Table 3 represents the operative and pathologic characteristics of patients who required a re-operation due to adnexal lesion. The origin of the adnexal lesion was confirmed as the ovary in 59 (83%) patients, and as the peritoneum in 8 (11.2%) patients during surgery. All of the adnexal lesions arising after hysterectomy and required a re-operation were benign; no malignancy was detected in our group of patients. Intraoperative and postoperative complications developed in 8 (11.2%) patients, including bowel injury in 5 patients, and urinary tract injury in 2 patients. Only 1 patient required a blood transfusion. The mean length of hospital stay was 4.1±2.8 (minimum 1, maximum 18) days. Table 4 presents data of 7 patients who required a second operation other than hysterectomy due to an adnexal lesion. The time interval between the diagnosis and second operation varied between 2-36 months, and all of the pathologies detected in these 7 patients were confirmed to be benign.

## Discussion

The present retrospective study investigating the adnexal lesions arising after hysterectomy indicates that almost half of the pathologies (48.1%) detected after surgery disappeared during follow-up period. The adnexal lesions that were re-operated were more symptomatic, larger, and more complicated lesions with more septations, mural nodules, and abnormal Doppler findings raising doubts of malignancy. However, all lesions were found to be benign, mostly originating from the ovary.

Women and physicians are faced with the decision of whether to remove or preserve ovaries and fallopian tubes during hysterectomy for benign indications. The number of bilateral salpingo-oophorectomies performed concomitantly with hysterectomy has been declining over the last 10 years, particularly among women aged under 55 years (3). The American Congress of Obstetricians and Gynecologists, in the practice bulletin reaffirmed in 2016, states that ‘strong consideration should be made for retaining normal ovaries’ (15). However, women with ovarian preservation are at risk for future oophorectomy (16). In a recent study of Casiano et al. (12), the incidence of oophorectomy after hysterectomy was found as 9.2% (12). They postulated that disruption of ovarian blood flow after hysterectomy might alter ovarian function, which could lead to adnexal pathologies.

Our study is one of the very few that focuses on the causes of adnexal lesions after hysterectomy. The study is also distinct in terms of the inclusion of a group of patients in whom adnexal lesions disappeared during the follow-up period. In 1996, Dekel et al. (6) in their cohort study of 2561 hysterectomies (during which one or both ovaries were preserved) over a period of 20 years found that the incidence of residual ovary syndrome was 2.85%. Residual ovary syndrome was described as a persistent pelvic mass presenting with pain, tenderness or dyspareunia in patients in whom at least one of the ovaries was preserved during hysterectomy. The most common reasons for subsequent oophorectomy were pain (71.3%) and the presence of an asymptomatic adnexal mass (24.6%). Holub et al. (10) examined the re-operation rates of adnexal lesions after different approaches of hysterectomy, namely abdominal, vaginal and laparoscopic approach, and found that the highest rate of reoperation was after abdominal hysterectomy (5.67%), followed by laparoscopic (3.18%) and vaginal approaches (0.69%). They suggested that the important factors affecting the reoperation rate were age, primary histologic findings, and smaller peritoneal trauma. In the study by Baloglu et al. (9), the reoperation rate due to secondary ovarian lesions after hysterectomy was found as 4.3%-3.8% for patients without oophorectomy and 5.9% for patients with unilateral oophorectomy. They concluded that women with unilateral oophorectomy at the time of hysterectomy had more than twice the risk of secondary ovarian lesions compared with those without oophorectomy at hysterectomy.

Recently, Shiber et al. (7) investigated adnexal masses requiring reoperation in women with previous hysterectomy with or without adnexectomy. They reported that the majority of adnexal masses requiring re-operation after hysterectomy were gynecologic in origin, benign, and arose from the ovary. In accordance with this study, all lesions that were re-operated in our study were benign and mostly originating from the ovary. Apart from others, the study of Shiber et al. (7) included a group of patients returning for surgery after hysterectomy and bilateral salpingectomy, although the number of patients was small. They argued that this small number of patients requiring re-operation after hysterectomy and bilateral salpingectomy might reflect a decreased risk for future surgery or may just indicate an insufficient time interval to evaluate the development of adnexal lesion. Recent studies have challenged the traditional concept of the pathogenesis of ovarian cancer, suggesting the fallopian tubes as the originating organ for the disease. In a recent population-based study by Falconer et al. (17), it was found that salpingectomy in benign indications was associated with reduced risk of ovarian cancer. They concluded that women scheduled for hysterectomy for benign indications should be informed about the risk-reducing effect of salpingectomy on ovarian cancer, and may be offered during common procedures such as hysterectomy. Our study did not include patients who had undergone hysterectomy and bilateral salpingectomy because the procedure has gained popularity and has been truly adopted in recent years. We will be able to evaluate this group of patients when sufficient time has passed with this emerging practice.

Different from other studies, we also evaluated morbidities during these repeat surgeries. Although we did not encounter any mortality, morbidities such as need for blood transfusion, urinary tract and bowel injury were seen in 8 of the 71 patients.

There are some limitations of our study. First, we could not give the incidence of adnexal lesions emerging after hysterectomy and re-operation rate due to these lesions because we could only evaluate women with adnexal mass after hysterectomy who were available for follow-up in our instution. Secondly, we did not have a group of patients with hysterectomy and bilateral salpingectomy during the years the study was conducted. However, in our current practice, we inform patients about prophylactic and opportunistic salpingectomy, and offer it as a preventing strategy for ovarian cancer in low-risk and high-risk patients, as well as for the prevention of benign pathologies.

In conclusion, despite its limitations, our study sheds light on guidance and information on surgical decisions for women presenting with adnexal lesion after hysterectomy. In our patients, almost half of the lesions arising after hysterectomy disappeared during follow-up, and all lesions that were re-operated were benign and mostly originating from the ovary. Patient counseling and the decision to perform a repeat operation due to an adnexal lesion after hysterectomy should be made on an individual basis.

## Figures and Tables

**Table 1:**
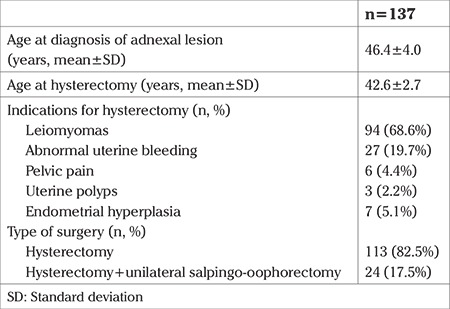
Age and hysterectomy details of patients

**Table 2:**
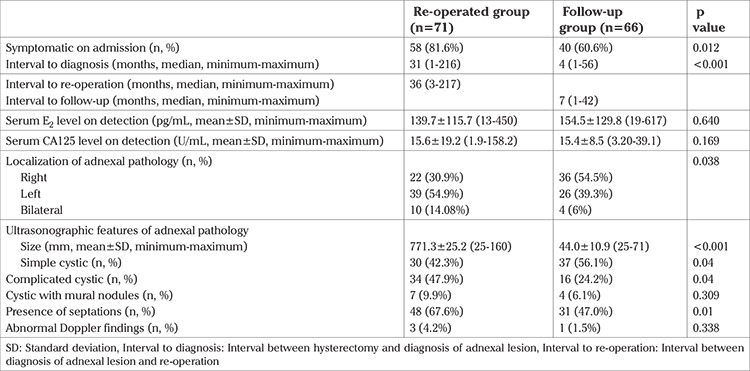
Characteristics of the adnexal pathology in the re-operated and the follow-up groups

**Table 3:**
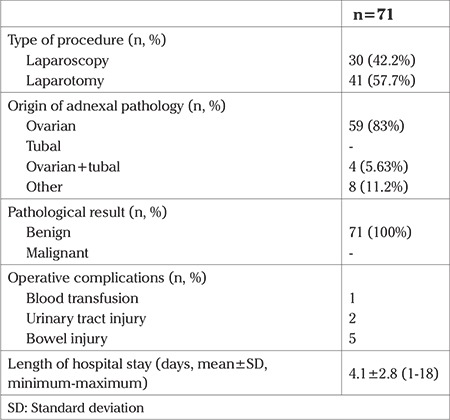
Operative and pathologic characteristics of patients who were re-operated due to adnexal lesion

**Table 4:**
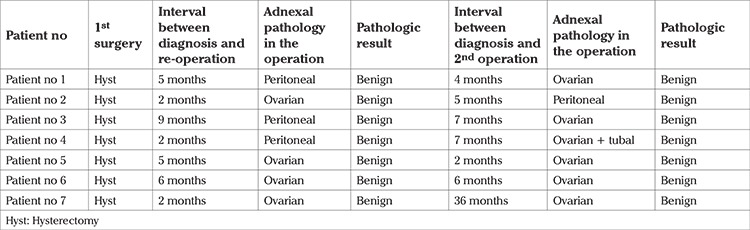
Patients who were operated for the second time for adnexal lesion after hysterectomy
